# KPC-2-producing *Klebsiella pneumonia*e in a hospital in the Midwest region of Brazil

**DOI:** 10.1590/S1517-838246246220140174

**Published:** 2015-06-01

**Authors:** Camila Arguelo Biberg, Ana Claudia Souza Rodrigues, Sidiane Ferreira do Carmo, Claudia Elizabeth Volpe Chaves, Ana Cristina Gales, Marilene Rodrigues Chang

**Affiliations:** 1 Universidade Federal de Mato Grosso do Sul, Laboratório de Pesquisas Microbiológicas, Universidade Federal de Mato Grosso do Sul, Campo Grande, MS, Brasil, Laboratório de Pesquisas Microbiológicas, Universidade Federal de Mato Grosso do Sul, Campo Grande, MS, Brazil.; 2 Hospital Regional de Mato Grosso do Sul, Campo Grande, MS, Brasil, Laboratório de Microbiologia do Hospital Regional Rosa Pedrossian de Mato Grosso do Sul, Campo Grande, MS, Brazil.; 3 Hospital Regional de Mato Grosso do Sul, Campo Grande, MS, Brasil, Comissão de Controle de Infecção Hospitalar, Hospital Regional Rosa Pedrossian de Mato Grosso do Sul, Campo Grande, MS, Brazil.; 4 Universidade Federal de São Paulo, Laboratório Alerta, Divisão de Doenças Infecciosas, Universidade Federal de São Paulo, São Paulo, SP, Brasil, Laboratório Alerta, Divisão de Doenças Infecciosas, Universidade Federal de São Paulo, São Paulo, SP, Brazil

**Keywords:** *Klebsiella pneumoniae*, carbapenems, drug-resistant bacteria

## Abstract

The emergence of β-lactamase-producing *Enterobacteriaceae* in the last few decades has become major challenge faced by hospitals. In this study, isolates of *Klebsiella pneumoniae* carbapenemase-2 (KPC-2)-producing *K. pneumoniae* from a tertiary hospital in Mato Grosso do Sul, Brazil, were characterized. Bacterial identification was performed by matrix-assisted laser desorption/ionization time-of-flight (MALDI-TOF; Bruker Daltonics, Germany) mass spectrometry. The minimum inhibitory concentrations of carbapenems were determined using the agar dilution method as recommended by the Clinical Laboratory Standards Institute guidelines. Carbapenemase production was detected using the modified Hodge test (MHT) and polymerase chain reaction (PCR), followed by DNA sequencing. Of 360 (12.2%) *K. pneumoniae* isolates obtained between May 2009 and May 2010, 44 (12.2%) were carbapenem nonsusceptible. Of these 44 isolates, thirty-six *K. pneumoniae* isolates that were positive by MHT and PCR carried the *bla* *_KPC-2_* gene. Thus, KPC-2producing *Klebsiella pneumonia*e has been present in a Brazilian hospital located in the Midwest region since at least 2009.

## Introduction

The increasing prevalence of bacterial resistance among *Enterobacteriaceae* isolated in hospitals is a global concern. The major mechanism of carbapenem resistance among these bacteria is the production of β-lactamase enzymes, including *Klebsiella pneumoniae* carbapenemase (KPC).

KPC-type enzymes inactivate β-lactam antibiotics, including cephalosporins, monobactams, and carbapenems, complicating the treatment of infections caused by these bacteria (Hirsch and Tam, 2010). KPC-2-producing *Enterobacteriaceae* have been isolated in many Brazilian medical centers, most frequently in teaching hospitals in the southern and southeastern regions. No data regarding the epidemiology of KPC strains in hospitals in the Brazilian Midwest are available (Nicoletti *et al.*, 2012).

The aim of this study was to investigate the presence of the *bla* *_KPC_* gene in *Klebsiella* spp. carbapenem-nonsusceptible isolates collected from a tertiary hospital in Mato Grosso do Sul, a Brazilian state in the Midwest region.

## Materials and Methods

### Bacterial isolates

*Klebsiella* spp. isolates that were nonsusceptible to imipenem, meropenem, and/or ertapenem were collected from hospitalized patients at the Regional Hospital of Mato Grosso do Sul (RHMS) between May 2009 and May 2010. The bacterial isolates were recovered from urine, blood, surgical wound exudates, catheter tips, tracheal aspirates and spinal cerebrospinal fluid samples. Surveillance cultures were not included. Microbiology lab-books and patient medical records were consulted to obtain demographic and clinical data.

### Identification and antimicrobial susceptibility

The *Klebsiella* spp. isolates were initially identified using conventional biochemical reactions at the RHMS clinical laboratory. Bacterial identification was confirmed by matrix-assisted laser desorption/ionization time-of-flight (MALDI-TOF) mass spectrometry using the Microflex LT System and analysis by Biotyper 2.0 software (Bruker Daltonics, Germany) at the Universidade Federal de São Paulo. The minimum inhibitory concentrations (MICs) of carbapenems were determined using the agar dilution method as recommended by the Clinical Laboratory Standards Institute (CLSI) guidelines (CLSI, 2011).

### Carbapenemase production

The modified Hodge test (MHT) with ertapenem and imipenem disks (10 μg each) was employed for the phenotypic detection of carbapenemase production (CLSI, 2011). A molecular investigation of the *bla* *_KPC_* gene was performed with all *K. pneumoniae* carbapenem nonsusceptible (resistant or intermediate) isolates.

DNA extraction, PCR and sequencing of the PCR products were performed according to Monteiro (2009) with minor modifications. DNA extraction was made by boiling method. One or two colonies were transferred to a microcentrifuge tube containing 300 μL of sterile MilliQ water. The suspension was boiled for 5 min and subsequently centrifuged for 1 min at 12,000 rpm. The supernatant was carefully aspirated and transferred to a new sterile microtube.

The following primers were used to amplify the bla_KPC_ gene: forward, 5′ TCGCTAAACTCGAACAGG 3′ and reverse, 5′ TTACTGCCCGTTGACGCCCAATCC 3′.

### PCR reaction

A master mix solution containing 1.0 μL of each primer (10 μmol), 12.5 μL of Go Taq ® Green Master Mix 2× (Promega, Madison, USA) and 8.5 μL of sterile MilliQ water was prepared. Then, 2 μL of DNA was added to achieve a final reaction volume of 25 μL. The reactions were amplified in an Eppendorf AG System, Eppendorf Mastercycler (Hamburg, Germany).

The cycling parameters were as follows: 10 min at 94 °C, followed by 35 cycles of denaturation at 94 °C for 1 min, annealing at 52 °C for 1 min, and extension at 72 °C for 1 min. The PCR amplification was completed with a final extension cycle at 72 °C for 10 min.

The PCR products were sequenced after purification using a QIA quick Gel Extraction kit (Qiagen, Hilden, Alemanha) as described by the manufacturer. The amplified genomic DNA was quantified by optical density in a spectrophotometer (NanoDrop® ND-1000 UV-Vis, version 3.2.1; Thermo Fisher Scientific, Wilmington, DE, USA). Approximately 70 ng of DNA was prepared for sequencing using the Big Dye Terminator Cycle Sequencing (Applied Biosystems, Foster City, USA) kit. Sequencing was performed on an ABI PRISM 3130 Genetic Analyzer (Applied Biosystems, Foster City, USA).

The resulting DNA sequences and their corresponding protein sequences were analyzed using the Lasergene Software Package (DNASTAR, Madison, WI) and compared with genetic databases available on the Internet (http://www.ebi.ac.uk/fasta33/ and http://www.ncbi.nlm.nih.gov/BLAST/).

This study was approved by the Research Ethics Committee of the Federal University of Mato Grosso do Sul.

## Results

During the study period, 360 isolates of *Klebsiella* spp. were identified by the RHMS clinical laboratory, of which 44 (12.2%) were nonsusceptible to carbapenems according to the CLSI breakpoints (CLSI, 2011). Identification as *Klebsiella pneumoniae* was confirmed by MALDI-TOF for all isolates. The antimicrobial susceptibility testing results for the carbapenems are reported in Table 1. Thirty-six of the forty-four carbapenem-nonsusceptible *K. pneumoniae* isolates were phenotypic carbapenemase producers as determined by the MHT, and all of those 36 isolates carried the *bla* *_KPC-2_* gene.

**Table 1:** *In vitro* antimicrobial activity of carbapenems against 44 *K. pneumoniae* isolates.

Antimicrobial agent	MIC (mg/L)		% by Category^1^	
		
	50%	90%	Susceptible	Nonsusceptible
Ertapenem	4	32	6.8	93.2
Meropenem	0.5	8	79.5	20.5
Imipenem	0.5	8	84.1	15.9

1 Breakpoint criteria established by the CLSI document (CLSI, 2011).

**Figure 1:**
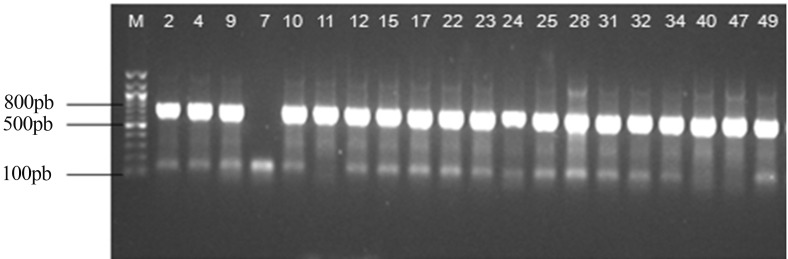
PCR amplification profile of the *bla* *_KPC_* gene (800 bp) from the *K.* pneumoniae isolates. M: 100 bp DNA ladder marker. Samples 2, 4, 9, 7, 10, 11, 12, 15, 17, 22, 23, 24, 25, 28, 31, 32, 34, 40, 47, and 49: *K.* pneumoniae clinical isolates*.* Sample 7 was negative for the presence of the *bla* *_KPC_* gene.

The PCR amplification profile of the *bla* *_KPC_* gene (800 bp) from the *K. pneumoniae* isolates is shown in Figure 1 *.*

Patients infected with KPC-producing *K. pneumoniae* were primarily admitted to the intensive care unit (ICU) (43%), followed by the internal medicine department and coronary care unit (13.6% each, respectively). The age of the patients ranged from 0 to 91 years, with a median age of 68 years. Of the 44 patients included in this study, 43.2% died. KPC-producing *K. pneumoniae* (36) was most frequently isolated from urine (41.7%), blood cultures (25%), surgical wound exudates (22.3%), catheter tips (5.5%) and tracheal aspirates (5.5%).

## Discussion

The prevalence of carbapenem-resistant Enterobacteriaceae has increased substantially during the last decade (Castanheira *et al.*, 2012; Nordmann *et al.*, 2011). The rapid increase and dissemination of carbapenemases, such as KPC, is a major challenge for clinical laboratories and physicians. The identification of the bacterial mechanisms of resistance is critical for infection control and epidemiological studies. However, molecular biology techniques for the detection of resistance genes are not yet available in most Brazilian routine laboratories.

In this study, we detected the presence of KPC-2-producing *K.* pneumoniae. This subtype is one of the most frequently occurring worldwide (Nadkarni *et al.*, 2009; Nordmann *et al.*, 2011), and the prevalence of this subtype in other Brazilian regions has been described previously (Castanheira *et al.*, 2012; Monteiro *et al.*, 2009; Nicoletti *et al.*, 2012). KPC-producing *K.* pneumoniae in Brazil was first described in 2006 by Monteiro *et al.* (2009) in a patient from the state of Pernambuco. An increasing number of cases were subsequently reported in geographically distant Brazilian cities, indicating the wide dissemination of KPC-2-producing isolates in Brazil (Castanheira *et al.*, 2012; Nicoletti *et al.*, 2012).

As shown in Table 1, resistance to ertapenem (MIC ≥ 1) was more frequent (93%) than resistance to imipenem (16%). These findings are in agreement with data reported by the Centers for Disease Control and Prevention (CDC) indicating that ertapenem resistance is the best marker for carbapenemase production (CDC, 2012). The MHT demonstrated accurate results, with 100% sensitivity and 100% specificity, compared with PCR, corroborating a study by Fehlberg *et al.* (2012).

In our study, eight *K. pneumoniae* isolates were KPC negative, suggesting the involvement of other resistance mechanisms. Carbapenem resistance may involve multiple mechanisms, such as production of carbapenemases (KPC, NDM, OXA, and MβL) alone or in combination with the loss of porins (Doumith *et al.*, 2009.), ESBL (TEM, SHV, CTX-M) and/or AmpC enzymes associated with porin loss, and the presence of efflux pumps (Carvalhaes *et al.*, 2009; Fehlberg *et al.*, 2012, Queenam *et al.*, 2007).

The high number of patients over 60 years of age (65.9%) and the high frequency of ICU admissions (43%) suggests that colonization by these multi-resistant bacteria is favored by the high number of invasive procedures and the prolonged use of broad-spectrum antibiotics associated with these units (Beirão *et al.*, 2011; Nordmann *et al.*, 2011).

A rapid and effective method for detecting KPC-producing *K. pneumoniae* is needed to avoid therapeutic failures and introduce measures to prevent and control the dissemination of these multi-resistant microorganisms (Hirsch and Tam, 2010).

Notably, 38.6% of the KPC-producing *K. pneumoniae* isolates were detected in urine cultures, and 31.8% were detected in blood cultures. These data confirm literature findings that *Klebsiella* spp. is an important causative agent of urinary tract infections in hospitalized patients (Beirão *et al.*, 2011).

Finally, we conclude that this study provides evidence of the presence of *K. pneumoniae* isolates carrying the *bla*_KPC-2_ gene in a Brazilian hospital located in the Midwest region since at least 2009. The results presented in this study further support the dissemination of this pathogen throughout the national territory. Understanding the mechanisms underlying resistance may facilitate the implementation of preventive measures, control of the dissemination of these pathogens, and the implementation of surveillance programs and effective therapies.
